# Range expansion of invasive shrubs: implication for crown fire risk in forestlands of the southern USA

**DOI:** 10.1093/aobpla/plw012

**Published:** 2016-02-22

**Authors:** Hsiao-Hsuan Wang, Carissa L. Wonkka, William E. Grant, William E. Rogers

**Affiliations:** 1 Department of Wildlife and Fisheries Sciences, Texas A&M University, College Station, TX 77843, USA; 2 Department of Ecosystem Science and Management, Texas A&M University, College Station, TX 77843, USA; 3 Present address: Department of Agronomy and Horticulture, University of Nebraska, Lincoln, NE 68583, USA

**Keywords:** Biological invasion, dispersal model, habitat quality, invasive plants, invasive species, logistic regression, zero-inflated negative binomial regression

## Abstract

Non-native plant invasions and changing management activities have dramatically altered the structure and composition of forests worldwide. Invasive shrubs and fire suppression have led to increased densification and biomass accumulation in forest ecosystems of the southeastern USA. Notably, Chinese and European privets are rapid growing, shade-tolerant shrubs which number among the most aggressive invasive species in these forests. Privet encroachment has caused losses of native diversity, alteration of ecosystem processes and changes in community structure. The latter has become manifest through decreases in fine herbaceous fuels concurrent with increases in coarse woody fuels in forest understoreys. These alterations in fuel structure will potentially lead to less frequent, but more severe forest fires, which threaten important forest resources during extreme weather conditions. Drawing on extensive data sets compiled by the US Forest Service, we integrated statistical forecasting and analytical techniques within a spatially explicit, agent-based, simulation framework to predict potential range expansion of Chinese and European privet (*Ligustrum sinense* and *L. vulgare*) and the associated increase in crown fire risk over the next two decades in forestlands of Mississippi and Alabama. Our results indicate that probability of invasion is positively associated with elevation, adjacency (within 300 m) to water bodies, mean daily maximum temperature, site productivity and private land ownership, and is negatively associated with slope, stand age, artificial regeneration, distance to the nearest road and fire disturbance. Our projections suggest the total area invaded will increase from 1.36 to ≈31.39% of all forestlands in Mississippi and Alabama (≈7 million hectares) and the annual frequency of crown fires in these forestlands will approximately double within the next two decades. Such time series projections of annual range expansions and crown fire frequency should provide land managers and restoration practitioners with an invasion chronology upon which to base proactive management plans.

## Abstract (precis)

Non-native plant invasions and changing management activities have altered the structure and composition of forests. Invasive shrubs and fire suppression have led to increased densification and biomass accumulation in forest ecosystems of the southeastern United States. Notably, the encroachment of non-native privets has altered ecosystem processes and caused changes in community structure. The latter has become manifest through decreases in fine herbaceous fuels concurrent with increases in coarse woody fuels in forest understories. These alterations in fuel structure will potentially lead to less frequent, but more severe forest fires, which threaten forest resources during extreme weather conditions.

## Introduction

Each year millions of acres of wildland unintentionally burn causing extensive property and resource losses. Annual wildfire suppression costs in the USA exceed $1 billion placing a considerable burden on state and federal taxpayers. Despite this substantial expenditure, 55 544 fires burned 9 159 917 acres in 2012 (NIFC 2012). Wildfires result in millions of dollars in property damage, substantial loss of natural resources, especially timber and wildlife, and potential human death and injury. In addition, wildfires that deviate from the historic fire regime of the ecosystem with respect to frequency or intensity can disrupt a variety of critical ecosystem functions and jeopardize the long-term integrity of an affected area. Facilitation of undesirable species invasions, threats to the persistence of endangered species and increases in soil erosion and attendant water quality are all potential consequences of fire regime alteration (D'Antonio *et al*. 2009; Smith *et al*. 2011). For instance, stand-replacing crown fires have been occurring more frequently in forests with historical low-intensity, frequent fire regimes because of fire suppression and fuel accumulation, causing disruption of the production of essential ecosystem services (Dale *et al*. 2001).

Southeastern forests are one of the areas under the highest fire danger in the USA. In most years, the south leads the USDA Forest Service regions in number of wildfires (Gaither *et al*. 2011). Although these fires are usually smaller in acreage and overall damage than fires in the Western USA because of forest fragmentation and accessibility to firefighters, some of the largest fires in the USA have occurred in this region during periods of extreme drought (Marshall *et al*. 2008).

Understorey shrubs are often the most hazardous fuels (Stephens and Ruth 2005), and they can act as ladder fuels to carry surface fires into the forest canopy. In Western forests, Thompson and Spies (2010) found the greatest crown damage in areas with high shrub cover, despite lower fine fuels. Similarly, Andreu *et al*. (2012) found that reducing shrubs in many types of southeastern forests consistently resulted in reduced fire flamelengths. Crown fires kill trees, destroying valuable timber and increase the suppression efforts necessary to contain the wildfire (Albini and Stocks 1986; Michaletz and Johnson 2007). Forest understoreys have become increasingly thicketized in southeastern US forests due to the introduction and proliferation of non-native shrub species. Little experimental data exist regarding the effect of this thicketization on the frequency of crown fires in this region.

Exotic shrub invasions of southeastern US forests have been shown to significantly alter fire regimes (Mack and D'Antonio 1998; Brooks *et al*. 2004). Invasive shrubs grow rapidly and substantially increase the live, coarse woody fuel loads in forest understoreys. Live woody fuels can inhibit or increase fire spread and intensity depending on moisture content (Varner *et al*. 2005). During normal weather conditions for the Southeast, this increased shrub density reduces fire risk by reducing fine herbaceous fuels and increasing moisture content of foliage litter below the shrub layer (Thaxton and Platt 2006; Nowacki and Abrams 2008). However, during extreme drought conditions, this thicketized understorey can increase fire intensity as the shrubs experience lowered moisture content and full or partial crown dieback, increasing coarse woody fuel loading (Van Wilgen and Richardson 1985; Grace 1998). These elevated fire intensities increase the likelihood of a devastating crown fire occurring. This is especially concerning given predictions of increases in summer and fall fire danger and longer fire seasons in the southeastern USA as a result of climate change in upcoming decades (Liu *et al*. 2010; Mitchell *et al*. 2014). These dense shrub thickets also increase vertical continuity within the forest, which reduces the intensity needed for crown fire ignition since crown fire ignition is a function of height between surface fuels and forest crown (Van Wagner 1977).

**Figure 1.**
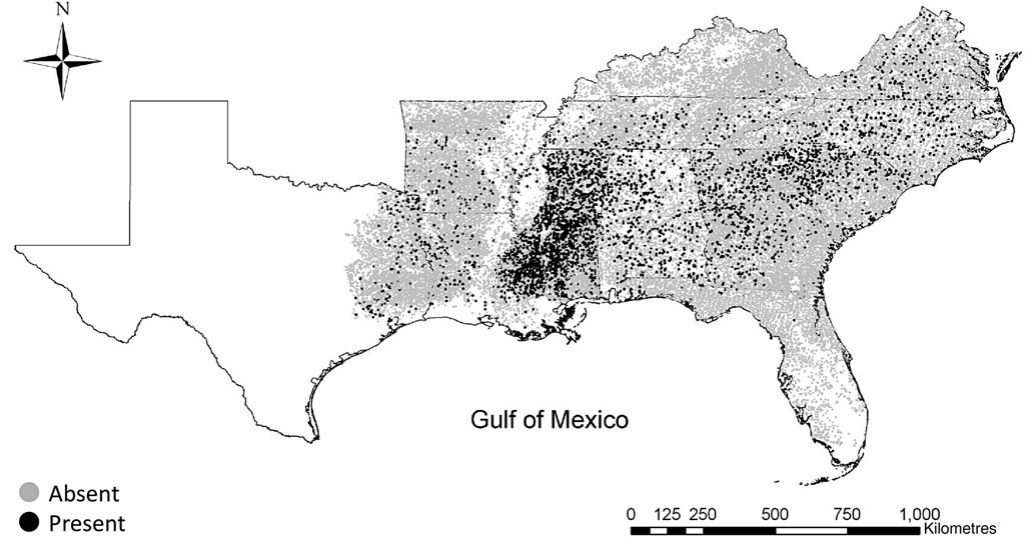
Current distribution of exotic privets in forestlands of the southern USA (USDA 2013). Gray and black dots indicate the absence and presence of exotic privets, respectively.

Based on the Forest Inventory and Analysis (FIA) surveys from early 2000 until December 2012, there were a total of 42 637 forested plots in the southern USA (USDA 2012). Sixty-six major invasive exotic plant species have been detected on forestlands in Alabama, Arkansas, Florida, Georgia, Kentucky, Louisiana, North Carolina, South Carolina, Tennessee, east Texas and Virginia (USDA 2013). Among these are many shrubs and vines that cause thicketization of the understorey. Chinese and European privet (*Ligustrum sinense* and *L. vulgare*) (henceforth exotic privets) are among the most aggressive invasive shrubs in this region, having invaded 9.64 % of forestlands, primarily in Alabama and Mississippi (Fig. 1).

Exotic privets form dense understorey stands spreading vegetatively to fill forest gaps. These uniform thickets greatly increase vertical continuity in southern forests, reaching over 20 m in height at times (Zammit and Westoby 1987). Observational and limited experimental evidence suggests that the dense woody understorey created by exotic privet invasion can supress fine herbaceous fuels and lower fire risk during periods of high foliar moisture. However, during drought, dense understorey shrub thickets with higher dead-to-live fuel ratios could greatly increase available fuels and can lead to more intense and severe fires than the historically frequent, but less intense surface fires of uninvaded forests (Brockway and Lewis 1997; Varner *et al*. 2005). Aditionally, thick midstorey strata can catch fallen pine needles, potentially increasing the flamability of these course woody fuels (Brockway and Lewis 1997). More reliable predictions of the speed and scope of exotic privet range expansion and associated crown fire threats are critically needed to improve the management of these invasions and their potential effects on crown fire risk. Yet such predictions remain a challenge due to modelling and data limitations. In this article, we first describe an approach for predicting potential range expansion of exotic privets in forestlands of Alabama and Mississippi. The approach integrates statistical forecasting and analytical techniques within a spatially explicit, agent-based, simulation framework. We then examine the potential effect of exotic privets on crown fire risk. Finally, we identify where new invasions are most likely to occur, and forecast the geographical extent of range expansion and the associated crown fire frequency over the next two decades.

## Methods

### Target ecosystem and focal species

Chinese and European privets are multi-stemmed, shade-tolerant, semi-evergreen to evergreen perennial shrubs. European privet was introduced to the USA in the mid-1800s and Chinese privet was introduced in 1852 and both were planted widely in the south as ornamental landscaping hedges (Haragan 1996). Both species have escaped cultivation and are presently naturalized from Florida to New England and as far west as Kansas and eastern Texas (Dirr 2011). They grow to a maximum height of 7–10 m within a few years of germination and colonize by sprouting from the roots or seed germination (Dirr 2011). As many as 1300 fleshy fruits per square metre of canopy are produced annually (Zammit and Westoby 1987; Strong *et al*. 2005). The seeds are widely dispersed by birds and mammals, have high viability and few germination requirements (Dirr 2011). Both species are capable of sustained rapid growth in low light and nutrient poor conditions which, consequently, allows them to readily displace native shrub and herbaceous species and form dense understorey stands, especially along roadsides and riparian corridors (Harrington and Miller 2005; Webster *et al*. 2006).

We focussed our investigation on the woodlands of Alabama and Mississippi because of the extent of privet invasion into these areas. Alabama and Mississippi have a humid, subtropical climate with 1400 mm of precipitation on average and an average temperature of 18 °C. The growing season can reach as many as 300 days per year in the southern portions of the states. Alabama and Mississippi are part of the southern region of the USA, providing vast timber resources (McNulty *et al*. 2000). Privet invasions occur in all forest types in these states, including longleaf-slash pine, loblolly shortleaf pine, oak-hickory hardwoods and oak-pine communities (USDA 2012, 2013).

### Model description

To predict the future range expansion of exotic privets and the associated effects on crown fire risk in forestlands of Alabama and Mississippi, we developed a spatially explicit, agent-based, invasion model following the general procedure described by Wang *et al*. (2011, 2012). The model consisted of a grid of 17 360 geo-referenced cells (agents), each representing a 2428-ha (6000 acres) plot of land, which is the size of the sampling units in the national array of permanent sampling areas maintained by the US Forest Service (FIA) (USDA 2005). The basic sampling design of FIA consists of a lattice of 2428-ha hexagons, with one sample plot located randomly within each hexagon (USDA 2005). We assigned each cell to one of seven land types (urban/built-up land, agricultural land, rangeland, forest land, water, wetland or barren land) based on land use and land cover data (USGS 2009). To each of these forest land cells (2742 in Alabama and 3770 in Mississippi), we assigned additional characteristics based on the FIA data set (USDA 2012), including landscape features, forest conditions, management activities and disturbances, and climatic conditions, as well as the current frequency of crown fires **[see Supporting Information—Appendix S1]**. We also assigned to each cell the percentage of land currently occupied by exotic privets, which we based on the Southern Nonnative Invasive Plant data Extraction Tool (USDA 2013). We merged the various data sets using ArcMap™ 10 (ESRI, Redlands, CA, USA) and imported the data associated with each cell into VB.NET© (Microsoft, 2003), where each cell was described by the indicated characteristics as well as by rules governing its dynamics. We then ran 240 twenty-year Monte Carlo simulations.

**Figure 2.**
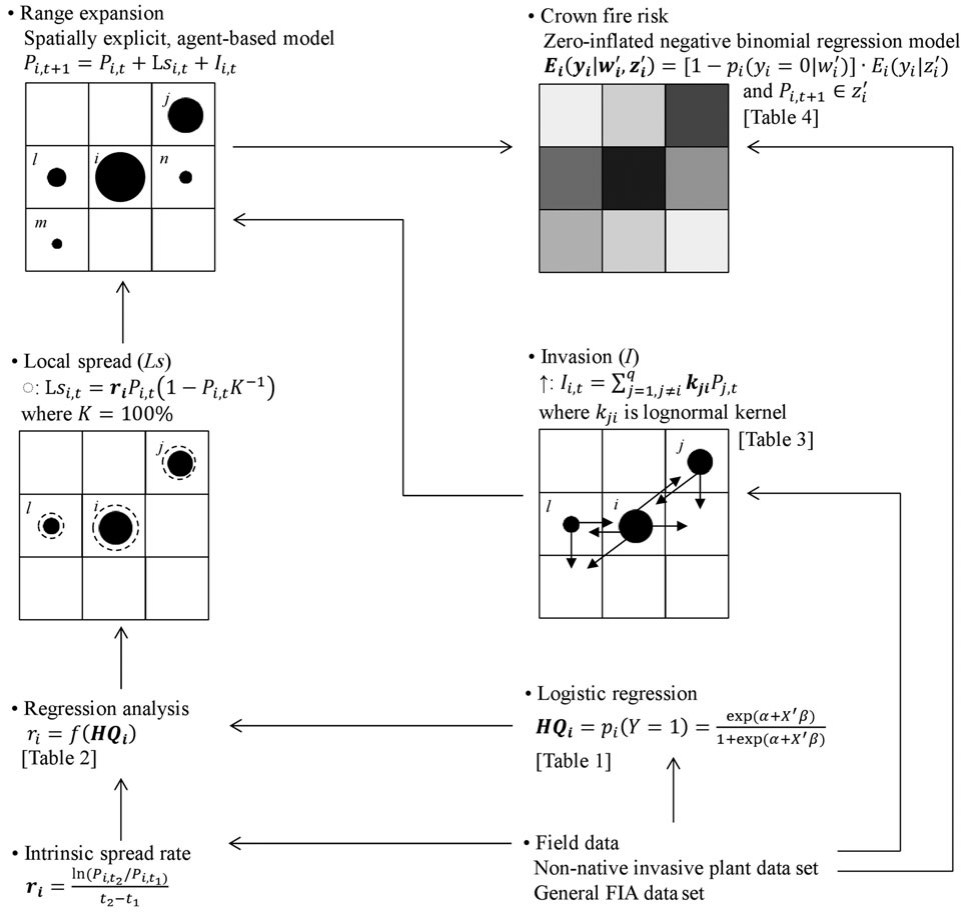
Diagrammatic representation of the linkages among field data, statistical forecasting methodologies and analytical techniques within a spatially explicit, agent-based, simulation framework to predict potential range expansion and associated crown fire risk pattern of exotic privets. Arrows represent the flow of model development. Bold letters represent parameters that were estimated (HQ*_i_*, *r_i_*, *E_i_*) or calibrated (*k_ji_*) based on field data.

We represented annual changes in terms of the percentage of land occupied by exotic privets in each cell (Δ*P_i_*_,*t*_/Δ*t*) as resulting from local spread within a cell (Ls*_i,t_*) plus invasion from other cells (*I_i,t_*):

(1) $$P_{i,t+1}=P_{i,t}+\text{L}{\text{s}}_{i,t}+I_{i,t}$$

where

(2) $$\text{L}{\text{s}}_{i,t+1}=\text{L}{\text{s}}_{i,t}+r_{i}P_{i,t}(1-P_{i,t}K_{i}^{-1})$$

and

(3) $$I_{i,t+1}=I_{i,t}+\overset{q}{\underset{\;j=1,j\neq i}{\sum }}k_{\;ji}P_{\;j,t}$$

where *P_i_*_,*t*_ is the percentage of land occupied by exotic privets in cell *i* at time *t*. Ls*_i_*_,*t*_ is the local spread in terms of the percentage of land occupied by exotic privets within cell *i* at time *t*, which has a logistic growth form. *I_i_*_,*t*_ is the increase of the percentage of land occupied by exotic privets due to invasion from adjacent cells to cell *i* at time *t*, which represents the dispersal process. *r_i_* is the mean intrinsic rate of local spread within cell *i* and *K_i_* is the carrying capacity within cell *i*, both represented in terms of the percentage of land occupied by exotic privets. *k_ji_* is the proportion of a lognormal dispersal kernel representing invasion from cell *j* to cell *i* represented in terms of the percentage of land occupied by exotic privets (Fig. 2). We set *P_i_*_,0_ equal to that reported for the year 2003 and assumed all *K_i_* = 100 based on information in the Southern Nonnative Invasive Plant data Extraction Tool, which indicates that exotic privets already occupy over 95 % of some plots (USDA 2013).

We calculated *r_i_* based on available data from the FIA data set (USDA 2012) and Southern Nonnative Invasive Plant data Extraction Tool (USDA 2013) following the method described in Wang *et al*. (2011):

(4) $$r_{i}=\frac{\text{ln}(P_{i,t_{2}}/P_{i,t_{1}})}{t_{2}-t_{1}}$$

where $\;P_{i,t_{1}}$ and $P_{i,t_{2}}$ are the percentage of land occupied by exotic privets in cell *i* at the year of the first (*t*_1_) and second (*t*_2_) survey, respectively. We excluded plots that had been subjected to any site preparation treatments or invasion control or if $P_{i,t_{1}}$ was greater than $P_{i,t_{2}}$. The FIA non-native invasive plant survey began in 2001 and is intended to survey one-fifth of the plots in each of 13 southern states annually (Rudis *et al*. 2006). At the time of our analysis, the first cycle of surveys had been completed for both Alabama and Mississippi, and the second cycle, which began in 2006, had been completed for Alabama, but only 3 % of second cycle had been completed for Mississippi. For those plots for which the second cycle data were not yet available, we estimated *r_i_* as a function of a habitat quality index, HQ*_i_* (0 ≤ HQ*_i_* ≤ 1, calculation described below), since the leading edges of invasive species expansion most often are correlated with habitat quality (Jarnevich and Stohlgren 2009). We explored the relationship between *r_i_* and HQ*_i_* using several regression analyses including linear (*r_i_* = *a* + *b*HQ*_i_*), logarithmic (*r_i_* = *a* + *b* ln HQ*_i_*), power $\left(r_{i}=a+b{\text{HQ}}_{i}^{-1}\;\text{and}\;r_{i}=a+b{\text{HQ}}_{i}^{2}\right)$ and exponential $\left(r_{i}=a\;\text{exp}(b{\text{HQ}}_{i}^{0.5}),\;\;r_{i}=a\;\text{exp}(b\text{H}{\text{Q}}_{i})\;\text{and}r_{i}=a\;\text{exp}(b{\text{HQ}}_{i}^{2})\right)$ (Gámez-Virués *et al*. 2010; Wang *et al*. 2011). We identified the best-fit equation based on *P*-value of estimated coefficients and *R*^2^.

We calculated HQ*_i_* for each of the 6512 forested plots sampled during the first survey cycle in terms of invasion probability using logistic regression (Agresti 2007):

(5) $$\text{H}{\text{Q}}_{i}=\;p_{i}(Y=1)=\frac{\text{exp}(\alpha +{X^{\prime }}_{i}\beta )}{\left[1+\text{exp}(\alpha +{X^{\prime }}_{i}\beta )]=f\;(\alpha +{X^{\prime }}_{i}\beta \right)}$$

where *Y* is a binary variable taking the value of either 1 if exotic privets are present or 0 otherwise, *p_i_*(*Y* = 1) is the probability for *Y* = 1 and means plot *i* is invaded by exotic privets, $X_{i}^{\prime }$ is the vector of climatic conditions, forest conditions and landscape features of plot *i*, and *α* and *β* (a vector) are coefficients. We identified the conditions and features to be tested following Lemke and Brown (2012) and Wang and Grant (2012, 2014), and selected the best equation for HQ*_i_* based on the Akaike information criterion (AIC) (Akaike 1973). In addition, we used the Hosmer–Lemeshow test to verify the statistical validity of the model (Hosmer and Lemeshow 2000).

We estimated *k_ji_* using a lognormal dispersal kernel, which has been used successfully to approximate observed dispersal patterns for a number of trees with animal-dispersed seeds (Stoyan and Wagner 2001; Greene *et al*. 2004; Russo *et al*. 2006; Wang *et al*. 2011):

(6) $$k_{\;ji}\approx \int \left[\frac{2\pi D}{{\left(2\pi \right)}^{1.5}SD^{2}}\text{exp}\left\{-\frac{{\left[\text{ln}(D/L)\right]}^{2}}{2S^{2}}\right\}\right]\;\text{d}D$$

where *k_ji_* represents the probability of dispersal from cell *j* to cell *i*, the integration variable *D* is the distance between cells *i* and *j*, *S* is the shape parameter, which we assumed was equal to 1, as is typically done when modelling woody species (Greene *et al*. 2004) and *L* is the scale parameter, which represents the dispersal velocity (in grid-width units per year) (Cannas *et al*. 2003). We parameterized several versions of the dispersal kernel function based on different hypothesized dispersal velocities ranging from 4927, 2463, 1642, … 704 m year^−1^ (grid cell width = 4927 m), that is, with *L* ranging from 1, 1/2, 1/3, … 1/7. For each value of *L*, we generated simulated invasion patterns by running 240 eight-year Monte Carlo simulations (Ott and Longnecker 2001) with the model initialized to represent the distribution pattern of exotic privets observed in the field in 2003 (USDA 2012). We identified the best value of *L* (hence, the best estimates of *k_ji_*) based on the likelihood comparison and spatial correlation as indicated by Mantel's test (Mantel 1967) using 999 randomizations and *α* = 0.05 level of significance between simulated and observed invasion patterns in the year of the second survey.

We estimated the expected frequency of crown fires in each cell (*E_i_*) by correlating observed frequencies of crown fires during a 5-year period with landscape features, forest conditions and climatic conditions **[see Supporting Information—Appendix S2]** using zero-inflated negative binomial regression:

(7) $$E_{i}(y_{i}|x_{i}^{\prime },z_{i}^{\prime })=[1-\;p_{i}(y_{i}=0|w_{i}^{\prime })]E_{i}(y_{i}|z_{i}^{\prime })$$

where $E_{i}(y_{i}|w_{i}^{\prime },\;z_{i}^{\prime })$ is the estimated mean frequency of crown fires during a 5-year period (*y_i_* > 0) in cell *i*; $\;p_{i}(y_{i}=0|w_{i}^{\prime })=\text{exp}(\gamma +\delta w_{i}^{\prime })/[1+\text{exp}(\gamma +\delta w_{i}^{\prime })]$ is the probability that the absence of crown fire was due to temperature and precipitation, and $w_{i}^{\prime }$ is the vector of climatic conditions; $E_{i}(y_{i}|z_{i}^{\prime })=\text{exp}(\epsilon +\zeta z_{i}^{\prime })$ is a negative binomial model predicting crown fire frequency and $z_{i}^{\prime }$ is the vector of landscape features and forest conditions, including percentage of land occupied by exotic privets; *γ* and, *δ*, *ε*, and *ζ* are vectors and estimated coefficients for the respective zero-inflated and negative binomial portions of the model. We selected the best-fit model that included all significant (*P*-value < 0.05) variables with lowest value of AIC (Akaike 1973). Crown fire frequency often has been related to landscape features (Hunter 1993; Keeling *et al*. 2006), forest conditions (Link *et al*. 2006; Keeley 2009) and climatic conditions (Litschert *et al*. 2012; Liu *et al*. 2012). Zero-inflated negative binomial regression commonly is used to adjust for count variables with excessive zeros (crown fires were absent from cells characterized by low temperature and high precipitation).

## Results

**Figure 3.**
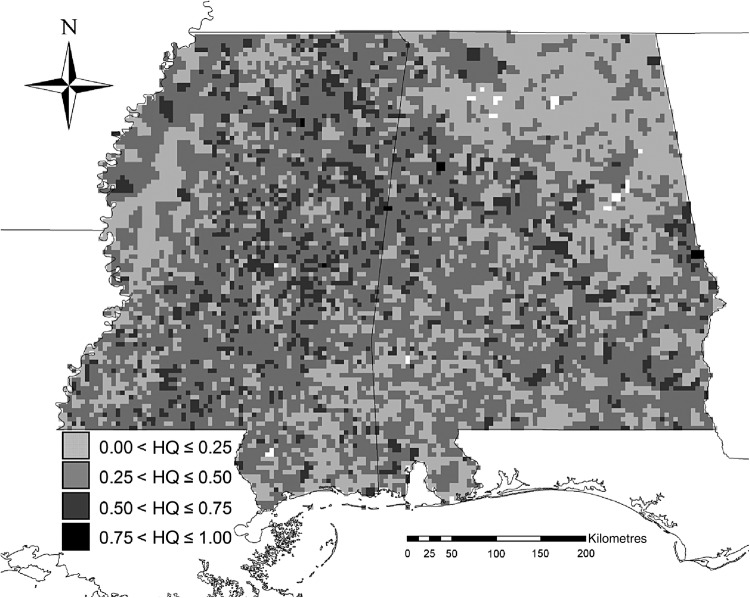
Estimated habitat quality (high values of HQ = high quality, estimated HQ values ranged from <0.01 to 0.95) for exotic privets in forestlands of Alabama and Mississippi.

Results of logistic regression indicated that habitat quality within cells (HQ*_i_*) was positively associated with adjacency (within 300 m) to water bodies, mean daily maximum temperature, site productivity and private land ownership, and was negatively associated with slope, stand age, artificial regeneration, distance to the nearest road and fire disturbance (Table 1). The logistic regression model correctly classified 66 % of the cells with regard to presence or absence of exotic privets, and the *P*-value of the Hosmer–Lemeshow test (0.5815) indicated no significant difference (*P*-value < 0.05) between observed and model-predicted occupancy values. HQ*_i_* values ranged from <0.01 to 0.95 and generally decreased from west to east (Fig. 3); these findings are similar to those of Wang and Grant (2012, 2014). Based on the best-fit equation (*R*^2^ = 0.72) relating the mean intrinsic spread rate within cells to habitat quality (*r_i_* = 0.3815 exp(0.8611HQ*_i_*), Table 2), the most favourable habitat (HQ = 0.95) produced a local spread rate (*r*) of 0.86. Of the various dispersal velocities we evaluated, only a velocity 985 m year^−1^ (*L* = 1/5 the width of a grid cell per year) produced simulated invasion patterns that were not statistically significantly different (*P*-value < 0.05) from the observed pattern (Table 3).

**Table 1.** Landscape features, climatic conditions, forest conditions and management activities and disturbances **[Supporting Information—Appendix S1]** selected, based on the indicated results of logistic regression, to estimate habitat quality (HQ = probability of invasion) for exotic privets on forested plots in Alabama and Mississippi. ^1^The estimated odds ratio indicates the change in the probability of invasion by exotic privets that would result from a one-unit change in the value of the indicated variable. For example, a one-unit increase in the site productivity signifies that invasion is 1.388 times more likely than before, after controlling for the other variables.

Variable	Estimated coefficient	Odds ratio^1^	*P*-value
Slope	−0.0512	0.950	<0.0001
Adjacency to water bodies within 300 m	0.2595	1.296	0.0006
Mean daily maximum temperature	0.9295	2.533	<0.0001
Stand age	−0.0083	0.992	<0.0001
Site productivity	0.3281	1.388	<0.0001
Artificial regeneration	−0.2323	0.793	<0.0001
Distance to the nearest road	−0.1950	0.823	<0.0001
Fire disturbance	−0.4267	0.653	0.0182
Forestland ownership	1.1145	3.048	<0.0001
Constant	−8.2364	–	<0.0001

**Table 2.** Comparison of several functional relationships between the mean intrinsic spread rate of exotic privets within cells (*r_i_*) and the index of habitat quality (HQ*_i_*), including linear (*r_i_* = *a* + *b*HQ*_i_*), logarithmic (*r_i_* = *a* + *b* ln HQ*_i_*), power $\left(r_{i}=a+b{\text{HQ}}_{i}^{-1}\;\mathrm{and}\;r_{i}=a\;+b{\text{HQ}}_{i}^{2}\right)$ and exponential $\left(r_{i}=a\;\text{exp}(b\text{H}{\text{Q}}_{i})\;\mathrm{and}\;r_{i}=a\;\text{exp}(b{\text{HQ}}_{i}^{2})\right)$. *P-values of all coefficients were <0.01.

Equation*	*R*^2^
*r_i_* = 0.2508 + 0.3877HQ*_i_*	0.41
*r_i_* = 0.9499 + 0.9351 ln HQ*_i_*	0.56
$r_{i}=0.9085-0.0037{\text{HQ}}_{i}^{-1}$	0.54
$r_{i}=0.0782+0.5778{\text{HQ}}_{i}^{2}$	0.45
*r_i_* = 0.3815 exp(0.8611HQ*_i_*)	0.72
$r_{i}=0.4155\;\text{exp}(0.7581{\text{HQ}}_{i}^{2})$	0.61

**Table 3.** Comparisons, via spatial correlation (Mantel's test), of the observed invasion pattern for exotic privets with those simulated assuming each of several invasion velocities (m year^−1^); *L* is the scale parameter, which represents dispersal velocity in grid-width units per year.

Velocity	*L*	Mantel's *r*	*P*-value
4927.00	1	−0.0824	0.967
2463.50	1/2	−0.0659	0.956
1642.33	1/3	−0.0047	0.821
1231.75	1/4	0.0255	0.179
985.40	1/5	0.2347	0.016
821.17	1/6	0.1729	0.047
703.86	1/7	0.1280	0.072

The best-fit zero-inflated negative binomial regression indicated that slope, physiographic class and percentage of land occupied by exotic privets in 2003 were statistically significant predictors of the frequency of crown fires (Table 4). The expected frequency decreased by 4 % (1 − exp(−0.0405) = 0.04) for each 1° increase of slope. The expected frequencies for sites in the xeric and mesic physiographic classes were ∼2.14 and 2.04 times higher, respectively, than those expected for sites in the hydric physiographic class. The expected frequency was ∼1.05 times higher for every % increase of forestland occupied by exotic privets. Mean daily precipitation and mean daily maximum temperature were statistically significant predictors of excessive zeros (that is, predictors of conditions under which crown fires were unlikely to occur). The log odds of being an excessive zero would decrease by 0.13 for every °C increase of mean daily maximum temperature and would increase by 0.27 for every cm increase of mean daily precipitation.

**Table 4.** Landscape features, climatic conditions and forest conditions **[Supporting Information—Appendix S2]** selected to estimate frequency of crown fires on forested plots in Alabama and Mississippi based on results of the zero-inflated negative binomial regression analysis.

Variable	Estimated coefficient	Standard error	*P*-value
Zero-inflated			
Mean daily maximum temperature	−0.1287	0.0292	<0.0001
Mean daily precipitation	0.2679	0.0711	<0.0001
Negative binomial			
Slope	−0.0405	0.0100	<0.0001
Physiographic class: xeric sites	0.7621	0.1542	<0.0001
Physiographic class: mesic sites	0.7114	0.1429	<0.0001
Percentage of land occupied by exotic privets	0.0444	0.0103	<0.0001

**Figure 4.**
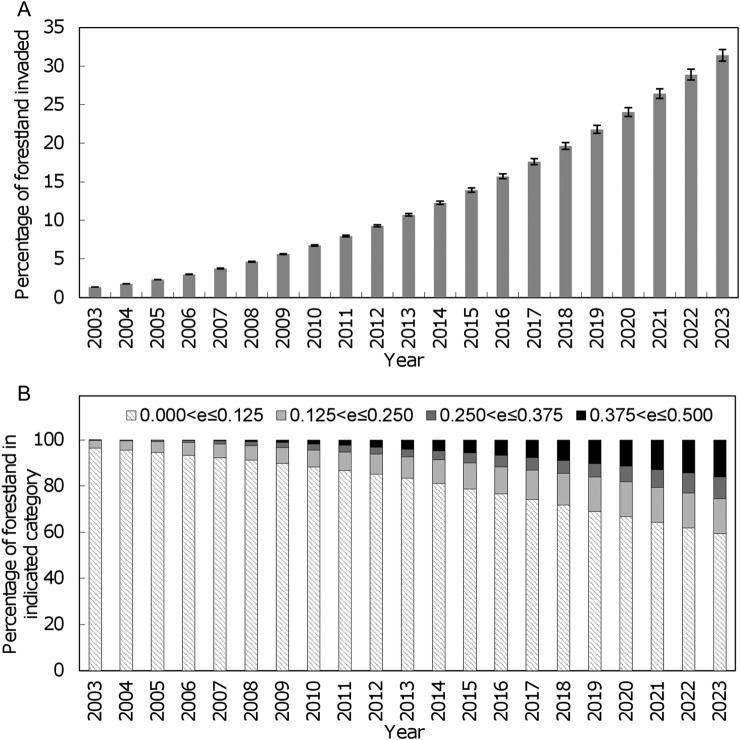
Simulated mean (±SE, *n* = 240) (A) percentage of forest land in Alabama and Mississippi invaded by exotic privets and (B) associated annual expected frequencies of crown fires on forest lands.

**Figure 5.**
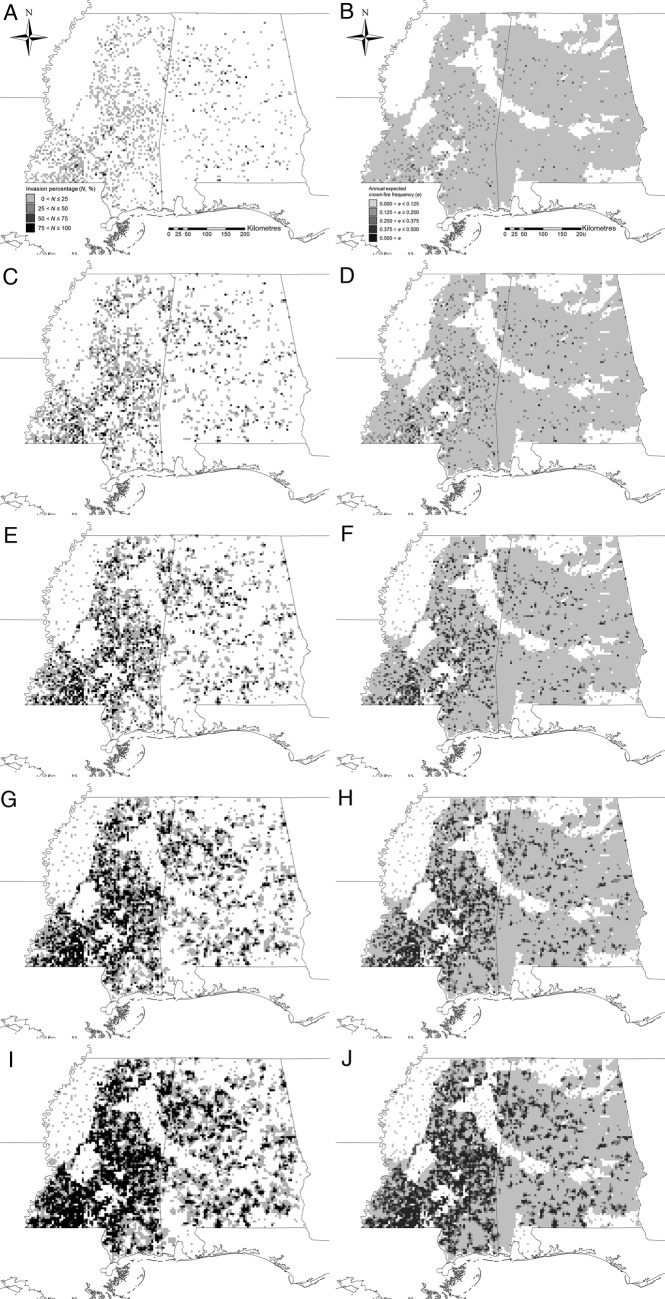
Typical simulated (A, C, E, G and I) invasion pattern of exotic privets in forest lands of Alabama and Mississippi and (B, D, F, H and J) associated annual expected frequencies of crown fires to the initial, 5th, 10th, 15th and 20th year, respectively.

Projections of future range expansion in forestlands of Mississippi and Alabama indicated that exotic privets have the potential to expand from the ≈0.3 million acres that they occupied in 2003 to ≈7 million acres in 2023 (Fig. 4A), which represents 31 % of all forestlands (≈22 million acres) within these two states. Geographically, projections suggested that the invasion has the potential to spread outward from virtually all of the relatively low-occupancy (<25 %) foci that were scattered throughout Alabama and Mississippi in 2003, the majority of which were located in south-western Mississippi, with occupancy levels increasing to >75 % in many areas by 2023 (Fig. 5A, C, E, G and I). The associated annual expected crown fire frequencies (*e_i_*) increased from only a few cells (312) in the 0.125 < *e_i_* ≤ 0.250 category in 2003 to 1391, 856 and 1479 cells in the 0.125 < *e_i_* ≤ 0.250, 0.250 < *e_i_* ≤ 0.375 and 0.375 < *e_i_* ≤ 0.500 categories, respectively, in 2023 (Figs 4B and 5B, D, F, H and J).

## Discussion

### Exotic privets ecology and crown fire risk

Our model results suggest a strong potential for greater crown fire risk throughout Alabama and Mississippi during extreme fire weather due to range expansion and increased density of exotic privets. High spread rates associated with the many areas of high habitat quality depicted by our model suggest that if current management practices persist, exotic privets could significantly spread into many unoccupied areas of Alabama and Mississippi and increase their dominance in already occupied areas. Our model, which is based on past occurrence of crown fire in the region, showed a positive correlation between privet occupation and crown fire risk, suggesting the potential for a greater number of crown fires in these states. Thicketization of understorey shrubs typically leads to forest mesophication in this region, reducing the frequency of fire because of the negative feedback between shrub cover and fine fuel loads and moisture content (Nowacki and Abrams 2008). However, under drought conditions, shrubs can experience dieback providing coarse woody fuels that carry fire despite discontinuous fine herbaceous fuel loading (Klos *et al*. 2009; Allen *et al*. 2010).

Increases in understorey shrub density have been shown to lead to hotter, more intense fires during extreme fire weather (Brockway and Lewis 1997; Varner *et al*. 2005; Duguy *et al*. 2013). Crown fires are more likely to become manifest with greater fire intensity because a larger break in vertical continuity can be overcome by hot fires with longer flame lengths than mild surface fires (Pollet and Omi 2002). In addition, understorey shrubs increase vertical continuity by providing ladder fuels by which surface fires ignite the crowns of mature forests (Doren and Whiteaker 1990; Grace 1998; Brooks *et al*. 2004; Stephens and Ruth 2005). Lonsdale (1993) showed that increased densities of the understorey shrub *Mimosa pigra* lead to an increased frequency of crown torching during wildfire. Similarly, Dibble and Rees (2005) showed that ladder fuels increased in areas invaded by privet and Schwilk *et al*. (2009) found that crown fires, although rare during low fire-danger weather, were more frequent in plots that had not been prescribed burned repeatedly in the past 40 years. The largest structural difference between the prescribed burn plots and the control plots was high levels of shrub cover (Schwilk *et al*. 2009). Despite potential for increased thicketization resulting from invasion to decrease fine herbaceous fuel, both pine and foliar litter, which have been shown to be the most important surface fuel in most southeastern forests (Ottmar and Prichard 2012), will still be available to carry surface fires in many forest types. Consequently, the moisture content of the shrub layer will determine whether it inhibits or increases the intensity of litter-driven surface fires (Agee 2002).

Despite observational evidence of low flammability in privet-invaded areas, little experimental evidence exists regarding privet stand flammability. Our data show that highly invaded stands in the study region have experienced crown fires. The FIA database used in our study included 220 plots that had a crown fire in the 5 years prior to data collection. Of those that had a crown fire, 45 plots were invaded by privet with an average privet cover of 49 %. Therefore, while probability of ignition could be low in privet stands, especially in years of average or above average precipitation, privet-invaded stands do burn, and our model results show that high privet invasion increases the probability of crown fire initiation during a fire.

The potential for climate change adds to the threat of crown fires posed by exotic privet invasion. Our model shows that average daily precipitation and maximum daily temperatures are significant predictors of conditions under which crown fires will be likely to occur. Crown fire risk increases with decreasing precipitation and crown fires are more likely to occur with higher daily maximum temperature. The southern USA is already one of the most fire-prone areas of the USA (Gaither *et al*. 2011). Drought is predicted to occur more frequently in some areas of the southeastern USA due to increased temperatures and attendant increases in evaporation as well as water loss from plants (USGCRP 2009; Mitchell *et al*. 2014). General Circulation Models show increases in average air temperature (Smith *et al*. 2009). In addition, average precipitation predictions are uncertain for this region. However, most models show great increases in the distribution and variability of precipitation and more frequent and intense drought events (Dale *et al*. 2001; Allen *et al*. 2010). This can greatly increase forest susceptibility to devastating wildfires. Indeed, most fires in the USA occur during periods of drought (Guyette *et al*. 2002), and fire hazard in the Southeast is predicted to increase under many climate change scenarios (Dale *et al*. 2001; Liu *et al*. 2010).

The humid semi-tropic region accumulates large fuel loads during rainy periods and hence poses an enormous fire risk during dry seasons (Dale *et al*. 2001; Schoennagel *et al*. 2005). Variability of precipitation has been shown to increase ubiquitously with most climate change prediction scenarios (Smith *et al*. 2009). While exotic privet invasions can reduce fire risk by eliminating surface fuels in some areas, and replacing flammable fuels (ericaceous species) with a less flammable woody understorey, the reduction in frequency of low-intensity surface fires only exacerbates the potential for devastating high-intensity fires as woody plants build up in the understorey, creating high loads of coarse woody fuels when shrub canopies dry out and die back during droughts (Stephens and Ruth 2005; Mandle *et al*. 2011). Moreover, elevated atmospheric CO_2_ levels have been shown to increase privet biomass and branching (Smith *et al*. 2008) potentially creating negative feedbacks that amplify the thicketization of forest understoreys and magnify catastrophic crown fire risks during extreme fire weather.

### Implications for exotic privet management

Using the output from our model, land owners and forest managers will be able to more accurately predict the extent and speed of potential exotic privet invasion over the next several decades. This will allow them to better focus their efforts aimed at reducing additional negative consequences associated with further privet encroachment. Landowners can apply preventative management in uninvaded areas that are likely to become invaded in the future while restoration efforts can be focussed in areas that are at a high risk for crown fire. Exotic privets have altered ecosystem function and suppressed native species regeneration and diversity in South Carolina (Kittell 2011), North Carolina (Merriam and Feil 2002) and Georgia (Wilcox and Beck 2007). Privet encroachment has the potential to exacerbate economic risks associated with timber losses caused by crown fires as climate change increases variability in precipitation (Murphy *et al*. 2014; Enright *et al*. 2015) and frequency of extreme drought. Exotic privets survive fire by sprouting from the root crown in response to damage of aboveground tissue and thus can quickly occupy the understorey of forest stands (Faulkner *et al*. 1989). Moreover, the risks wildfires pose to property and other economic capital, human health and safety, and a variety of ecological resources make it imperative that management action plans be implemented to mitigate the potential consequences of unimpeded invasion of southern forests by exotic privets.

There are many options available to effectively manage exotic privets and even eradicate it from an invaded area. Cutting and herbicide application has been used to effectively remove exotic privets (Wang and Grant 2014), and although an expensive method of eradication, this effective management tool might be appropriate in areas of high crown fire risk, with the costs of the management offset by the numerous potential economic losses associated with a wildfire. Additionally, while single applications of prescribed fire did not decrease privet abundance in one study (Caspary and Affolter 2012), repeated burns have been shown to control privet encroachment (Huebner 2005). Prescribed fires have been used effectively and relatively inexpensively to reduce fuel loads, especially loads of ladder and surface fuels (Agee and Skinner 2005; Stephens and Ruth 2005). However, under future climate predictions, increases in drought frequency could lead to more restrictions on prescribed burning in the southeastern USA (Mitchell *et al*. 2014). This could limit the temporal window for privet management via prescribed fire in this region. Open burning regulations that allow exemption from burn bans for certified prescribed burn managers should be considered for states in the southeastern USA in order to provide more opportunity for managers to meet invasive species management goals under an increasingly variable precipitation regime (Wonkka *et al*. 2015). Of course, managers need to know where these management strategies will be most needed and most effective because management of privet throughout the forestlands of Alabama and Mississippi is not logistically feasible due to high labour requirements and other associated costs of control. The output of our model should be tremendously beneficial in this regard.

## Conclusions

To move beyond reactive control efforts towards more proactive management of invasive species and associated crown fire requires prediction of potential ranges of invasive species on spatial scales relevant to land owners, forest managers and restoration practitioners. We drew upon extensive geo-reference data sets on invasive plants and crown fire records to develop a model predicting possible range expansion of Chinese and European privets and associated crown fire frequency. Our results suggest that the total area invaded will increase in the forestlands of Mississippi and Alabama, and the annual frequency of crown fires in these forestlands will approximately double within the next two decades. Such time series projections of annual range expansions and crown fire frequency should provide land owners, forest managers and restoration practitioners with an invasion chronology upon which to base proactive management plans.

## Contributions by the Authors

All authors shared in collecting data, constructing the model and writing.

## Conflict of Interest Statement

None declared.

## Supporting Information

The following additional information is available in the online version of this article —

**Appendix S1.** Descriptions, values or units of measure, and means and ranges or frequencies of landscape features, forest conditions and management activities and disturbances evaluated as potential determinants of site invasion by exotic privets in Alabama and Mississippi.

**Appendix S2.** Descriptions, values or units of measure, and means and ranges or frequencies of landscape features, climatic conditions and forest conditions evaluated as potential determinants of crown fire frequency on forested plots in Alabama and Mississippi.

Additional Information

## References

[PLW012C1] Agee JK 2002 The fallacy of passive management managing for firesafe forest reserves. Conservation in Practice 3:18–26. 10.1111/j.1526-4629.2002.tb00023.x

[PLW012C2] Agee JK, Skinner CN 2005 Basic principles of forest fuel reduction treatments. Forest Ecology and Management 211:83–96. 10.1016/j.foreco.2005.01.034

[PLW012C3] Agresti A 2007 An introduction to categorical data analysis. Hoboken, NJ: John Wiley and Sons, Inc.

[PLW012C4] Akaike H 1973 Information theory and an extension of the maximum likelihood principle. In: Kotz S, Johnson NL, eds. Second international symposium on information theory. Budapest: Academia Kiado, 267–281.

[PLW012C5] Albini FA, Stocks BJ 1986 Predicted and observed rates of spread of crown fires in immature Jack pine. Combustion Science and Technology 48:65–76. 10.1080/00102208608923884

[PLW012C6] Allen CD, Macalady AK, Chenchouni H, Bachelet D, Mcdowell N, Vennetier M, Kitzberger T, Rigling A, Breshears DD, (Ted) Hogg EH, Gonzalez P, Fensham R, Zhang Z, Castro J, Demidova N, Lim J-H, Allard G, Running SW, Semerci A, Cobb N 2010 A global overview of drought and heat-induced tree mortality reveals emerging climate change risks for forests. Forest Ecology and Management 259:660–684. 10.1016/j.foreco.2009.09.001

[PLW012C7] Andreu AG, Shea D, Parresol BR, Ottmar RD 2012 Evaluating fuel complexes for fire hazard mitigation planning in the southeastern United States. Forest Ecology and Management 273:4–16. 10.1016/j.foreco.2011.06.040

[PLW012C8] Brockway DG, Lewis CE 1997 Long-term effects of dormant-season prescribed fire on plant community diversity, structure and productivity in a longleaf pine wiregrass ecosystem. Forest Ecology and Management 96:167–183. 10.1016/S0378-1127(96)03939-4

[PLW012C9] Brooks ML, D'Antonio CM, Richardson DM, Grace JB, Keeley JE, Ditomaso JM, Hobbs RJ, Pellant M, Pyke D 2004 Effects of invasive alien plants on fire regimes. BioScience 54:677–688. 10.1641/0006-3568(2004)054[0677:EOIAPO]2.0.CO;2

[PLW012C10] Cannas SA, Marco DE, Páez SA 2003 Modelling biological invasions: species traits, species interactions, and habitat heterogeneity. Mathematical Biosciences 183:93–110. 10.1016/S0025-5564(02)00213-4 12604137

[PLW012C11] Caspary M, Affolter J 2012 Using prescribed burning to restore granite rock outcrop ecotones in the Piedmont of the southeastern United States. Ecological Restoration 30:228–236. 10.3368/er.30.3.228

[PLW012C12] Dale VH, Joyce LA, Mcnulty S, Neilson RP, Ayres MP, Flannigan MD, Hanson PJ, Irland LC, Lugo AE, Peterson CJ, Simberloff D, Swanson FJ, Stocks BJ, Michael Wotton B 2001 Climate change and forest disturbances. Climate change can affect forests by altering the frequency, intensity, duration, and timing of fire, drought, introduced species, insect and pathogen outbreaks, hurricanes, windstorms, ice storms, or landslides. BioScience 51:723–734. 10.1641/0006-3568(2001)051[0723:CCAFD]2.0.CO;2

[PLW012C13] D'Antonio CM, Chambers JC, Loh R, Tunison JT 2009 Applying ecological concepts to the management of widespread grass invasions. In: Inderjit, ed. Management of invasive weeds. Invading Nature—Springer series in invasion ecology The Netherlands: Springer, 123–149.

[PLW012C14] Dibble AC, Rees CA 2005 Does the lack of reference ecosystems limit our science? A case study in nonnative invasive plants as forest fuels. Journal of Forestry 103:329–338.

[PLW012C15] Dirr MA 2011 Dirr's encyclopedia of trees and shrubs. Portland, OR: Timber Press, Inc., 952 pp.

[PLW012C16] Doren RF, Whiteaker LD 1990 Effects of fire on different size individuals of *Schinus terebinthifolius*. Natural Areas Journal 10:107–113.

[PLW012C17] Duguy B, Paula S, Pausas JG, Alloza JA, Gimeno T, Vallejo RV 2013 Effects of climate and extreme events on wildfire regime and their ecological impacts. In: Navarra A, Tubiana L, Duguy B, Paula S, Pausas J, Alloza JA, Gimeno T,Vallejo R, eds. Regional assessment of climate change in the Mediterranean. Advances in global change research The Netherlands: Springer, 101–134.

[PLW012C18] Enright NJ, Fontaine JB, Bowman DMJS, Bradstock RA, Williams RJ 2015 Interval squeeze: altered fire regimes and demographic responses interact to threaten woody species persistence as climate changes. Frontiers in Ecology and the Environment 13:265–272. 10.1890/140231

[PLW012C19] Faulkner JL, Clebsch EEC, Sanders WL 1989 Use of prescribed burning for managing natural and historic resources in Chickamauga and Chattanooga National Military Park, USA. Environmental Management 13:603–612. 10.1007/BF01874966

[PLW012C20] Gaither CJ, Poudyal NC, Goodrick S, Bowker JM, Malone S, Gan J 2011 Wildland fire risk and social vulnerability in the southeastern United States: an exploratory spatial data analysis approach. Forest Policy and Economics 13:24–36. 10.1016/j.forpol.2010.07.009

[PLW012C21] Gámez-Virués S, Gurr GM, Raman A, Nicol HI 2010 Plant diversity and habitat structure affect tree growth, herbivory and natural enemies in shelterbelts. Basic and Applied Ecology 11:542–549. 10.1016/j.baae.2010.02.011

[PLW012C22] Grace JB 1998 Can prescribed fire save the endangered coastal prairie ecosystem from Chinese tallow invasion? Endangered Species UPDATE 15:70–76.

[PLW012C23] Greene DF, Canham CD, Coates KD, Lepage PT 2004 An evaluation of alternative dispersal functions for trees. Journal of Ecology 92:758–766. 10.1111/j.0022-0477.2004.00921.x

[PLW012C24] Guyette RP, Muzika RM, Dey DC 2002 Dynamics of an anthropogenic fire regime. Ecosystems 5:472–486.

[PLW012C25] Haragan PD 1996 Privet (*Ligustrum vulgare*, *L. sinense*, *L. japonicum*). In: Randall JM, Marinelli J, eds. Invasive plants: weeds of the global garden. Brooklyn, NY: Brooklyn Botanic Garden, 58–59.

[PLW012C26] Harrington TB, Miller JH 2005 Effects of application rate, timing, and formulation of glyphosate and triclopyr on control of Chinese privet (*Ligustrum sinense*). Weed Technology 19:47–54. 10.1614/WT-03-220R2

[PLW012C27] Hosmer DW, Lemeshow S 2000 Applied logistic regression. New York, NY: John Wiley and Sons, Inc.

[PLW012C28] Huebner CD 2005 Fire and invasive exotic plant species in eastern oak communities: an assessment of current knowledge. Proceedings of the Fire in eastern oak forests: delivering science to land managers Gen. Tech. Rep. NRS-P-1, 218–232.

[PLW012C29] Hunter ML Jr 1993 Natural fire regimes as spatial models for managing boreal forests. Biological Conservation 65:115–120. 10.1016/0006-3207(93)90440-C

[PLW012C30] Jarnevich CS, Stohlgren TJ 2009 Temporal management of invasive species. In: Inderjit, eds. Management of invasive weeds. Dordrecht, The Netherlands: Springer, 103–122.

[PLW012C31] Keeley JE 2009 Fire intensity, fire severity and burn severity: a brief review and suggested usage. International Journal of Wildland Fire 18:116–126. 10.1071/WF07049

[PLW012C32] Keeling EG, Sala A, Deluca TH 2006 Effects of fire exclusion on forest structure and composition in unlogged ponderosa pine/Douglas-fir forests. Forest Ecology and Management 237:418–428. 10.1016/j.foreco.2006.09.064

[PLW012C33] Kittell MM 2011 Relationships among invasive Chinese privet, plant diversity, and small mammal captures in southeastern deciduous forests. MS Thesis, Clemson University, Clemson, SC, 35 pp.

[PLW012C34] Klos RJ, Wang GG, Bauerle WL, Rieck JR 2009 Drought impact on forest growth and mortality in the southeast USA: an analysis using Forest Health and Monitoring data. Ecological Applications 19:699–708. 10.1890/08-0330.1 19425432

[PLW012C35] Lemke D, Brown JA 2012 Habitat modeling of alien plant species at varying levels of occupancy. Forests 3:799–817. 10.3390/f3030799

[PLW012C36] Link SO, Keeler CW, Hill RW, Hagen E 2006 *Bromus tectorum* cover mapping and fire risk. International Journal of Wildland Fire 15:113–119. 10.1071/WF05001

[PLW012C37] Litschert SE, Brown TC, Theobald DM 2012 Historic and future extent of wildfires in the Southern Rockies Ecoregion, USA. Forest Ecology and Management 269:124–133. 10.1016/j.foreco.2011.12.024

[PLW012C38] Liu Y, Stanturf J, Goodrick S 2010 Trends in global wildfire potential in a changing climate. Forest Ecology and Management 259:685–697. 10.1016/j.foreco.2009.09.002

[PLW012C39] Liu Y, Goodrick SL, Stanturf JA 2012 Future U.S. wildfire potential trends projected using a dynamically downscaled climate change scenario. Forest Ecology and Management. 10.1016/j.foreco.2012.06.049.

[PLW012C40] Lonsdale WM 1993 Rates of spread of an invading species—*Mimosa pigra* in northern Australia. Journal of Ecology 81:513–521. 10.2307/2261529

[PLW012C41] Mack MC, D'Antonio CM 1998 Impacts of biological invasions on disturbance regimes. Trends in Ecology and Evolution 13:195–198. 10.1016/S0169-5347(97)01286-X 21238260

[PLW012C42] Mandle L, Bufford JL, Schmidt IB, Daehler CC 2011 Woody exotic plant invasions and fire: reciprocal impacts and consequences for native ecosystems. Biological Invasions 13:1815–1827. 10.1007/s10530-011-0001-3

[PLW012C43] Mantel N 1967 The detection of disease clustering and a generalized regression approach. Cancer Research 27:209–220. 6018555

[PLW012C44] Marshall DJ, Wimberly M, Bettinger P, Stanturf J 2008 Synthesis of knowledge of hazardous fuels management in loblolly pine forests. Asheville, NC: USDA Forest Service, Southern Research Station.

[PLW012C45] Mcnulty SG, Moore JA, Iverson L, Prasad A, Abt R, Smith B, Sun G, Gavazzi M, Bartlett J, Murray B, Mickler RA, Aber JD 2000 Application of linked regional scale growth, biogeography, and economic models for southeastern United States pine forests. World Resource Review 12:298–320.

[PLW012C46] Merriam RW, Feil E 2002 The potential impact of an introduced shrub on native plant diversity and forest regeneration. Biological Invasions 4:369–373. 10.1023/A:1023668101805

[PLW012C47] Michaletz ST, Johnson EA 2007 How forest fires kill trees: a review of the fundamental biophysical processes. Scandinavian Journal of Forest Research 22:500–515. 10.1080/02827580701803544

[PLW012C48] Mitchell RJ, Liu Y, O'brien JJ, Elliott KJ, Starr G, Miniat CF, Hiers JK 2014 Future climate and fire interactions in the southeastern region of the United States. Forest Ecology and Management 327:316–326. 10.1016/j.foreco.2013.12.003

[PLW012C49] Murphy BP, Bradstock RA, Boer MM, Carter J, Cary GJ, Cochrane MA, Fensham RJ, Russell-Smith J, Williamson GJ, Bowman DMJS 2014 Fire regimes of Australia: a pyrogeographic model system. Journal of Biogeography 40:1048–1058. 10.1111/jbi.12065

[PLW012C50] NIFC. 2012 Wildland Fire Statistics. National Interagency Fire Center. http://www.nifc.gov/fireInfo/fireInfo_statistics.html (13 January 2013).

[PLW012C51] Nowacki GJ, Abrams MD 2008 The demise of fire and “mesophication” of forests in the eastern United States. BioScience 58:123–138. 10.1641/B580207

[PLW012C52] Ott RL, Longnecker MT 2001 An introduction to statistical methods and data analysis. Pacific Grove, CA: Thomson Learning.

[PLW012C53] Ottmar RD, Prichard SJ 2012 Fuel treatment effectiveness in forests of the upper Atlantic Coastal Plain—an evaluation at two spatial scales. Forest Ecology and Management 273:17–28. 10.1016/j.foreco.2011.09.040

[PLW012C54] Pollet J, Omi PN 2002 Effect of thinning and prescribed burning on crown fire severity in ponderosa pine forests. International Journal of Wildland Fire 11:1–10. 10.1071/WF01045

[PLW012C55] Rudis VA, Gray A, Mcwilliams W, O'brien R, Olson C, Oswalt S, Schulz B 2006 Regional monitoring of nonnative plant invasions with the Forest Inventory and Analysis program. Proceedings of the Sixth Annual FIA Symposium Gen. Tech. Rep. WO-70, 49–64.

[PLW012C56] Russo SE, Portnoy S, Augspurger CK 2006 Incorporating animal behavior into seed dispersal models: implications for seed shadows. Ecology 87:3160–3174. 10.1890/0012-9658(2006)87[3160:IABISD]2.0.CO;2 17249240

[PLW012C57] Schoennagel T, Veblen TT, Romme WH, Sibold JS, Cook ER 2005 ENSO and PDO variability affect drought-induced fire occurrence in Rocky Mountain subalpine forests. Ecological Applications 15:2000–2014. 10.1890/04-1579

[PLW012C58] Schwilk DW, Keeley JE, Knapp EE, Mciver J, Bailey JD, Fettig CJ, Fiedler CE, Harrod RJ, Moghaddas JJ, Outcalt KW, Skinner CN, Stephens SL, Waldrop TA, Yaussy DA, Youngblood A 2009 The national Fire and Fire Surrogate study: effects of fuel reduction methods on forest vegetation structure and fuels. Ecological Applications 19:285–304. 10.1890/07-1747.1 19323191

[PLW012C59] Smith HG, Sheridan GJ, Lane PNJ, Nyman P, Haydon S 2011 Wildfire effects on water quality in forest catchments: a review with implications for water supply. Journal of Hydrology 396:170–192. 10.1016/j.jhydrol.2010.10.043

[PLW012C60] Smith JB, Schneider SH, Oppenheimer M, Yohe GW, Hare W, Mastrandrea MD, Patwardhan A, Burton I, Corfee-Morlot J, Magadza CHD, Füssel H-M, Pittock AB, Rahman A, Suarez A, Van Ypersele J-P 2009 Assessing dangerous climate change through an update of the Intergovernmental Panel on Climate Change (IPCC) “reasons for concern”. Proceedings of the National Academy of Sciences of the USA 106:4133–4137. 10.1073/pnas.0812355106 19251662 PMC2648893

[PLW012C61] Smith KE, Runion GB, Prior SA, Price AJ, Rogers HH, Torbert HA 2008 Chinese privet (*Ligustrum sinense*) in an elevated CO_2_ environment. Botany Research Journal 1:43–48.

[PLW012C62] Stephens SL, Ruth LW 2005 Federal forest-fire policy in the United States. Ecological Applications 15:532–542. 10.1890/04-0545

[PLW012C63] Stoyan D, Wagner S 2001 Estimating the fruit dispersion of anemochorous forest trees. Ecological Modelling 145:35–47. 10.1016/S0304-3800(01)00385-4

[PLW012C64] Strong CM, Brown DR, Stouffer PC 2005 Frugivory by wintering hermit thrush in Louisiana. Southeastern Naturalist 4:627–638. 10.1656/1528-7092(2005)004[0627:FBWHTI]2.0.CO;2

[PLW012C65] Thaxton JM, Platt WJ 2006 Small-scale fuel variation alters fire intensity and shrub abundance in a pine savanna. Ecology 87:1331–1337. 10.1890/0012-9658(2006)87[1331:SFVAFI]2.0.CO;2 16761611

[PLW012C66] Thompson JR, Spies TA 2010 Factors associated with crown damage following recurring mixed-severity wildfires and post-fire management in southwestern Oregon. Landscape Ecology 25:775–789. 10.1007/s10980-010-9456-3

[PLW012C67] USDA. 2005 The enhanced forest inventory and analysis program—national sampling design and estimation procedures. Asheville, NC: USDA Forest Service, Southern Research Station.

[PLW012C68] USDA. 2012 FIA data and tools. USDA Forest Service. http://www.fia.fs.fed.us/tools-data/default.asp (1 May 2013).

[PLW012C69] USDA. 2013 Southern Nonnative Invasive Plant data Extraction Tool (SNIPET). USDA Forest Service. http://srsfia2.fs.fed.us/data_center/index.shtml (1 May 2013).

[PLW012C70] USGCRP. 2009 Global climate change impacts in the United States. New York, NY: Cambridge University Press.

[PLW012C71] USGS. 2009 1:250,000 & 1:100,000 scale land use land cover (LULC). http://edc2.usgs.gov/geodata/index.php (16 April 2009).

[PLW012C72] Van Wagner C 1977 Conditions for the start and spread of crown fire. Canadian Journal of Forest Research 7:23–34. 10.1139/x77-004

[PLW012C73] Van Wilgen BW, Richardson DM 1985 The effects of alien shrub invasions on vegetation structure and fire behaviour in South African fynbos shrublands: a simulation study. Journal of Applied Ecology 22:955–966. 10.2307/2403243

[PLW012C74] Varner JM, Gordon DR, Putz FE, Hiers JK 2005 Restoring fire to long-unburned *Pinus palustris* ecosystems: novel fire effects and consequences for long-unburned ecosystems. Restoration Ecology 13:536–544. 10.1111/j.1526-100X.2005.00067.x

[PLW012C75] Wang H-H, Grant WE 2012 Determinants of Chinese and European privet (*Ligustrum sinense* and *Ligustrum vulgare*) invasion and likelihood of further invasion in southern U.S. forestlands. Invasive Plant Science and Management 5:454–463. 10.1614/IPSM-D-12-00038.1

[PLW012C76] Wang H-H, Grant WE 2014 Invasion of eastern Texas forestlands by Chinese privet: efficacy of alternative management strategies. Diversity 6:652–664. 10.3390/d6040652

[PLW012C77] Wang H-H, Grant WE, Swannack TM, Gan J, Rogers WE, Koralewski TE, Miller JH, Taylor JW Jr 2011 Predicted range expansion of Chinese tallow tree (*Triadica sebifera*) in forestlands of the southern United States. Diversity and Distributions 17:552–565. 10.1111/j.1472-4642.2011.00760.x

[PLW012C78] Wang H-H, Grant WE, Gan J, Rogers WE, Swannack TM, Koralewski TE, Miller JH, Taylor JW 2012 Integrating spread dynamics and economics of timber production to manage Chinese tallow invasions in southern U.S. forestlands. PLoS ONE 7:e33877 10.1371/journal.pone.0033877 22442731 PMC3307772

[PLW012C79] Webster CR, Jenkins MA, Jose S 2006 Woody invaders and the challenges they pose to forest ecosystems in the eastern United States. Journal of Forestry 104:366–374.

[PLW012C80] Wilcox J, Beck CW 2007 Effects of *Ligustrum sinense* Lour. (Chinese Privet) on abundance and diversity of songbirds and native plants in a southeastern nature preserve. Southeastern Naturalist 6:535–550. 10.1656/1528-7092(2007)6[535:EOLSLC]2.0.CO;2

[PLW012C81] Wonkka CL, Rogers WE, Kreuter UP 2015 Legal barriers to effective ecosystem management: exploring linkages between liability, regulations, and prescribed fire. Ecological Applications 25:2382–2393. 10.1890/14-1791.1 26910962

[PLW012C82] Zammit C, Westoby M 1987 Population structure and reproductive status of two Banksia shrubs at various times after fire. Vegetatio 70:11–20.

