# Supplementary material for: Range expansion of invasive shrubs: implication for crown fire risk in forestlands of the southern USA
Source: AoB Plants. 2016 Feb 22;8:plw012. doi: 10.1093/aobpla/plw012 (PMC4804204; doi:10.1093/aobpla/plw012)
Supplement: Additional Information [file supp_8_plw012_index.html]

Range expansion of invasive shrubs: implication for crown fire risk in forestlands of the southern United States — Range expansion of invasive shrubs: implication for crown fire risk in forestlands of the southern USA — Additional Information 

# Range expansion of invasive shrubs: implication for crown fire risk in forestlands of the southern USA

## Additional Information

Additional Information

- Supplementary file1 - docx file
- Supplementary file2 - docx file
