# Supplementary material for: Range expansion of invasive shrubs: implication for crown fire risk in forestlands of the southern USA
Source: AoB Plants. 2016 Feb 22;8:plw012. doi: 10.1093/aobpla/plw012 (PMC4804204; doi:10.1093/aobpla/plw012)
Supplement: Additional Information [file supp_plw012_plw012supp_file1.docx]

File 1

Appendix 1 Descriptions, values or units of measure, and means and ranges or frequencies of landscape features, forest conditions, and management activities and disturbances evaluated as potential determinants of site invasion by exotic privets in Alabama and Mississippi.

- Landscape features
- Elevation (continuous variable; measure unit: meters). Mean (range): 111 (-26~1366).
- Slope (continuous variable; measure unit: degrees). Mean (range): 4.37 (0~65).
- Adjacency to water bodies within 300 m (categorical variable; 1 means the cell *i* is adjacent to water bodies within 300 m; otherwise, 0). There are 1169 and 5343 cells representing 1 and 0, respectively.
- Climatic conditions
- Mean daily minimum temperature (continuous variable; measure unit: °C). Mean (range): 0.39 (-9.44~10.00).
- Mean daily maximum temperature (continuous variable; measure unit: °C). Mean (range): 34.37 (26.67~37.78).
- Forest conditions
- Stand age (continuous variable; measure unit: years). Mean (range): 34.48 (2~152).
- Site productivity (categorical variable, index of inherent capacity to grow commercial timber; 1, 2, 3, 4, 5, 6, and 7 mean the cell *i* has timber productivity of 0~1.39, 1.40~3.49, 3.50~5.94, 5.95~8.39, 8.40~11.54, 11.55~15.74, and >15.74 m^3^/ha-year, respectively. There are 11, 319, 1858, 2386, 1412, 482, and 42 cells representing 1, 2, 3, 4, 5, 6, and 7, respectively.
- Species diversity (continuous variable; Shannon’s species diversity index). Mean (range): 1.57 (0.01~3.00)
- Management activities and disturbances
- Timber harvest (categorical variable; 1 means timber has been harvested from the cell *i* within the past 5 years; otherwise, 0). There are 1664 and 4848 cells representing 1 and 0, respectively.
- Site preparation (categorical variable; clearing, slash burning, chopping, disking, bedding, or other practices clearly intended to prepare a site for regeneration; 1 means site preparation has occurred in the cell *i* within the past 5 years; otherwise, 0). There are 383 and 6129 cells representing 1 and 0, respectively.
- Artificial regeneration (categorical variable; planting or direct seeding has resulted in a stand at least 50 percent stocked with live trees of any size; 1 means artificial regeneration has occurred in the cell *i* within the past 5 years; otherwise, 0). There are 2653 and 3859 cells representing 1 and 0, respectively.
- Natural regeneration (categorical variable; growth of existing trees and/or natural seeding has resulted in a stand at least 50 percent stocked with live trees of any size; 1 means the cell *i* has been experienced natural regeneration within the past 5 years; otherwise, 0). There are 306 and 6206 cells representing 1 and 0, respectively.
- Distance to the nearest road (categorical variable; 1, 2, 3, 4, 5, 6, 7, 8, and 9 mean the distance between the cell *i* and the nearest road is <30, 30~90, 91~151, 152~304, 305~804, 805~1608, 1609~4827, 4828~8047, and >8047 meters, respectively). There are 301, 613, 632, 1205, 1882, 964, 280, 22, and 10 cells representing 1, 2, 3, 4, 5, 6, 7, 8, and 9, respectively.
- Insect disturbance (categorical variable; the area affected by insect disturbance must be at least 1 acre in size and a significant level of disturbance (mortality or damage to 25 percent of the trees in a stand) is required; 1 means the cell *i* has experienced insect disturbance within the past 5 years; otherwise, 0). There are 26 and 6486 cells representing 1 and 0, respectively.
- Disease disturbance (categorical variable; the area affected by disease disturbance must be at least 1 acre in size and a significant level of disturbance (mortality or damage to 25 percent of the trees in a stand) is required; 1 means the cell *i* has experienced disease disturbance within the past 5 years; otherwise, 0). There are 32 and 6480 cells representing 1 and 0, respectively.
- Fire disturbance (categorical variable; the area affected by fire disturbance must be at least 1 acre in size and a significant level of disturbance (mortality or damage to 25 percent of the trees in a stand) is required; 1 means the cell *i* has experienced fire disturbance within the past 5 years; otherwise, 0). There are 220 and 6292 cells representing 1 and 0, respectively.
- Animal (beavers, deer/ungulates, rabbits, domestic animals) disturbance (categorical variable; the area affected by animal disturbance must be at least 1 acre in size and a significant level of disturbance (mortality or damage to 25 percent of the trees in a stand) is required; 1 means the cell *i* has experienced animal disturbance within the past 5 years; otherwise, 0). There are 64 and 6448 cells representing 1 and 0, respectively.
- Wind (hurricanes and tornados) disturbance (categorical variable; the area affected by wind disturbance must be at least 1 acre in size and a significant level of disturbance (mortality or damage to 25 percent of the trees in a stand) is required; 1 means the cell *i* has experienced wind disturbance within the past 5 years; otherwise, 0). There are 456 and 6056 cells representing 1 and 0, respectively.
- Ownership
- Forestland ownership (categorical variable; 0 or 1 means the cell *i* is publicly owned or privately owned, respectively). There are 5913 and 599 cells representing 1 and 0, respectively.

Appendix 2 Descriptions, values or units of measure, and means and ranges or frequencies of landscape features, climatic conditions, and forest conditions evaluated as potential determinants of crown fire frequency on forested plots in Alabama and Mississippi.

- Landscape features
- Slope (continuous variable; measure unit: degrees). Mean (range): 4.37 (0~65).
- Physiographic class (categorical variable; 1, 2, and 3 represent the cell *i* belongs to xeric, mesic, and hydric sites, respectively; xeric sites - normally low or deficient in available moisture, mesic sites - normally moderate but adequate available moisture, hydric sites - normally abundant or overabundant moisture all year). There are 168, 6119, and 225 cells representing 1, 2, and 3, respectively.
- Climatic conditions
- Mean daily minimum temperature (continuous variable; measure unit: °C). Mean (range): 0.39 (-9.44~10.00).
- Mean daily maximum temperature (continuous variable; measure unit: °C). Mean (range): 34.37 (26.67~37.78).
- Mean daily precipitation (continuous variable; measure unit: cm). Mean (range): 10.05 (7.62~13.97).
- Forest conditions
- Stand age (continuous variable; measure unit: years). Mean (range): 34.48 (2~152).
- Percentage of land occupied by exotic privets (continuous variable; measure unit: %). Mean (range): 2.47 (0~95.45).
- Site productivity (categorical variable, index of inherent capacity to grow commercial timber; 1, 2, 3, 4, 5, 6, and 7 mean the cell *i* has timber productivity of 0~1.39, 1.40~3.49, 3.50~5.94, 5.95~8.39, 8.40~11.54, 11.55~15.74, and >15.74 m^3^/ha-year, respectively. There are 11, 319, 1858, 2386, 1412, 482, and 42 cells representing 1, 2, 3, 4, 5, 6, and 7, respectively.
