# Supplementary material for: Range expansion of invasive shrubs: implication for crown fire risk in forestlands of the southern USA
Source: AoB Plants. 2016 Feb 22;8:plw012. doi: 10.1093/aobpla/plw012 (PMC4804204; doi:10.1093/aobpla/plw012)
Supplement: Additional Information [file supp_plw012_plw012supp_file2.docx]

File 2

Appendix 2 Descriptions, values or units of measure, and means and ranges or frequencies of landscape features, climatic conditions, and forest conditions evaluated as potential determinants of crown fire frequency on forested plots in Alabama and Mississippi.

- Landscape features
- Slope (continuous variable; measure unit: degrees). Mean (range): 4.37 (0~65).
- Physiographic class (categorical variable; 1, 2, and 3 represent the cell *i* belongs to xeric, mesic, and hydric sites, respectively; xeric sites - normally low or deficient in available moisture, mesic sites - normally moderate but adequate available moisture, hydric sites - normally abundant or overabundant moisture all year). There are 168, 6119, and 225 cells representing 1, 2, and 3, respectively.
- Climatic conditions
- Mean daily minimum temperature (continuous variable; measure unit: °C). Mean (range): 0.39 (-9.44~10.00).
- Mean daily maximum temperature (continuous variable; measure unit: °C). Mean (range): 34.37 (26.67~37.78).
- Mean daily precipitation (continuous variable; measure unit: cm). Mean (range): 10.05 (7.62~13.97).
- Forest conditions
- Stand age (continuous variable; measure unit: years). Mean (range): 34.48 (2~152).
- Percentage of land occupied by exotic privets (continuous variable; measure unit: %). Mean (range): 2.47 (0~95.45).
- Site productivity (categorical variable, index of inherent capacity to grow commercial timber; 1, 2, 3, 4, 5, 6, and 7 mean the cell *i* has timber productivity of 0~1.39, 1.40~3.49, 3.50~5.94, 5.95~8.39, 8.40~11.54, 11.55~15.74, and >15.74 m^3^/ha-year, respectively. There are 11, 319, 1858, 2386, 1412, 482, and 42 cells representing 1, 2, 3, 4, 5, 6, and 7, respectively.
